# Supplementary material for: Associations of Mutually Exclusive Categories of Physical Activity and Sedentary Behavior with Body Composition and Fall Risk in Older Women: A Cross-Sectional Study
Source: Int J Environ Res Public Health. 2023 Feb 17;20(4):3595. doi: 10.3390/ijerph20043595 (PMC9961100; doi:10.3390/ijerph20043595)
Supplement: Supplementary file 1 [file ijerph-20-03595-s001.zip › ijerph-2189591-supplementary.pdf]

Table S1. Associations with body composition and fall risk assessment: Results from simple linear regression analysis

Outcome variables	Active-Low Sedentary		Active-High Sedentary		Inactive-Low Sedentary		Inactive-High Sedentary
	B (*SE*)	*p*	*B* (*SE*)	*p*	*B* (*SE*)	*p*	
Extracellular-to-intracellular water ratio (%)	-0.09 (1.2)	0.938	-0.64 (1.3)	0.624	-1. (0.99)	0.155	Reference
Extracellular-to-total body water ratio (%)	-0.01 (0.5)	0.992	-0.21 (0.6)	0.707	-0.60 (0.4)	0.153	Reference
Lean mass (kg)	0.14 (2.57)	0.956	1.96 (2.90)	0.500	0.34 (2.19)	0.878	Reference
Lean mass index (LMI, kg/m^2)	-0.79 (1.07)	0.458	0.02 (1.21)	0.988	-0.27 (0.91)	0.767	Reference
Fat mass (kg)	**-10.58 (3.67)**	**0.005**	-7.36 (4.14)	0.079	**-7.15 (3.13)**	**0.025**	Reference
Fat mass index (FMI, kg/m^2)	**-4.32 (1.34)**	**0.002**	-3.14 (1.51)	**0.041**	**-3.03 (1.14)**	**0.009**	Reference
Skeletal muscle mass (kg)	**3.75 (1.61)**	**0.022**	0.52 (1.82)	0.777	**3.23 (1.37)**	**0.021**	Reference
Skeletal muscle mass index (SMI, kg/m^2)	0.97 (0.50)	0.056	-0.12 (0.57)	0.836	**0.93 (0.43)**	**0.033**	Reference
Appendicular lean mass (kg)	**5.47 (1.86)**	**0.004**	0.28 (2.10)	0.893	1.94 (1.59)	0.224	Reference
Appendicular lean mass index (ALMI, kg/m^2)	**1.65 (0.60)**	**0.008**	-0.13 (0.68)	0.858	0.51 (0.52)	0.322	Reference

Appendicular fat mass (kg)	**-12.16 (4.21)**	**0.005**	-9.72 (4.76)	0.044	**-9.10 (3.59)**	**0.013**	Reference
Appendicular fat mass index (AFMI, kg/m^2)	**-2.23 (0.70)**	**0.002**	**-1.82 (0.79)**	**0.024**	**-1.70 (0.60)**	**0.006**	Reference
Perceived fall risk: fear of falling score	-2.35 (1.19)	0.052	-1.56 (1.38)	0.249	-0.47 (1.02)	0.642	Reference
Centre of pressure path length (cm)	-7.46 (4.48)	0.100	-7.30 (5.06)	0.153	-5.34 (3.82)	0.166	Reference
Sit-to-stand performance (reps)	**5.62 (1.83)**	**0.003**	2.77 (2.07)	0.183	**3.34 (1.56)**	**0.035**	Reference

Note: *B* = unstandardized regression coefficient, SE = standard error. Bold indicates $p < 0.05$.
